# Phylogeography of a herbal *Pinellia ternata* reveals repeated range expansions and inter/postglacial recolonization routes on the fragmented distribution pattern in China

**DOI:** 10.1002/ece3.70206

**Published:** 2024-08-29

**Authors:** Chunxue Jiang, Ming Wu, Shanshan He, Yuxia Lu, Cai Zhao

**Affiliations:** ^1^ Key Laboratory of Plant Resource Conservation and Germplasm Innovation in Mountainous Region (Ministry of Education) College of Life Sciences/Institute of Agro‐Bioengineering, Guizhou University Guiyang Guizhou Province China

**Keywords:** genetic diversity, glacial refugia, *P. ternata*, population structure, range expansion

## Abstract

Most plant phylogeographic studies in China have focused on the importance of genetic divergence and where should the shelter be located. Little attention has been paid to range expansion and recolonization routes in this region. In this study, two cpDNA fragments (*psb*K‐*psb*I and *trn*L‐F), two pairs of nuclear gene sequences (ITS and ETS), and nine pairs of SSR molecular markers were used, combined with Bayesian Skyline Plot method, gene barrier analysis, and species distribution models to explore the phylogeographical pattern, potential expansion routes and population dynamic history of *Pinellia ternata* from 22 population. The results showed that phylogeograhical pattern and genetic structure for *P. ternata* are effected by environmental heterogeneity and climate fluctuation, and it can be divided into two groups (Southwest group, Central and Eastern group) and thus there are at least two glacial refugia in China. Three expanding routes within groups were explored to contribute to the phylogeogrephic pattern of *P. ternata* based on the geographical distribution and network analysis of haplotypes. In a word, our study reveals repeated range expansions and inter/postglacial recolonization routes on the fragmented distribution pattern in China and resolves the refugia distributing in China and has also certain reference value for the protection of the medicinal plant *P. ternata*.

## Graphical abstract

Most plant phylogeographic studies in China have focused on the importance of genetic divergence and where should the shelter be located. Little attention has been paid to range expansion and recolonization routes in this region. Our study reveals repeated range expansions and inter/postglacial recolonization routes on the fragmented distribution pattern in China and resolves the refugia distributing in southern China and has also certain reference value for the protection of the medicinal plant *P. ternata*.

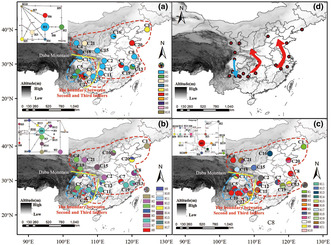

## 1 INTRODUCTION

Most living plant species in the Northern Hemisphere have experienced glacial–interglacial cycles over the past 2.5 million years. The most significant response of plants to Pleistocene climate fluctuations was a change in their range of distribution (Davis & Shaw, 2001), especially for species located in Europe and North America, where the development of huge ice sheets rendered large areas of Europe and North America inhospitable to vegetation (Hewitt, 1996, 2000; Park & Donoghue, 2019). During, species in these areas were driven southward, but a subsequent warm interglacial/postglacial period allowed these plants to expand northward (Zorrilla‐Azcué et al., 2021). This broad range of extension‐contraction (EC) models is more common in species in Europe and North America (Ni et al., 2010; Qiu et al., 2011), while there are few studies in China.

Ancient geological events (mountain uplift and volcanic eruptions) and climate changes (such as glacial/interglacial cycles) are important drivers of the evolution and genetic structure of plant species (Hewitt, 2004; Hickerson et al., 2010). At the same time, long‐term environmental heterogeneity along elevation or latitude gradients also contributes to genetic differentiation between species (Gugger et al., 2013). There is growing evidence that Quaternary climate oscillations have a large influence on the current distribution and genetic structure of species, which may vary by latitude and topography (Hewitt, 2000, 2003, 2011). Due to geographical barriers and climate fluctuations, the distribution patterns and population genetic structure of some species have been reshaped. In addition, population size, mating system, and biological characteristics of species also have important effects on the differentiation and evolutionary history of species populations (Stewart et al., 2010; Zhang, Li, Fritsch, et al., 2015; Zhang, Li, Liu, et al., 2015). For example, some studies have shown that the location of Quaternary glaciation plant refuges was largely determined by the species' adaptability to the external environment (Liao et al., 2015; Stewart et al., 2010; Wang et al., 2015). During the Quaternary glacial period, many species experienced extinction events due to repeated bottlenecks and genetic drift, leading to further differentiation, evolution, and genetic isolation within species (Hickerson et al., 2010; Keppel et al., 2012; Soltis et al., 2006; Stewart et al., 2010). The present population genetic structure of a species carries signals of past dynamics (Hewitt, 2000). Understanding the effects of orogeny, climate fluctuations, and environmental heterogeneity on spatial genetic patterns of species, especially on dominant vegetation species or endangered species, not only helps to reveal the complex evolutionary history of species but also helps to predict biological responses to future climate change (Jiang et al., 2018).

The strong uplift of the Tibetan Plateau is an important geological event that causes global climate change and stimulates the East Asian monsoon, making the merotropic zonality of Chinese flora increasingly obvious (An et al., 2001; Raymo & Ruddiman, 1992; Spicer et al., 2003). Therefore, the alternation of dry and wet climates has profoundly affected the distribution pattern and historical evolution of plants in China. Stable climate, mature plant communities, vast landscapes, high spatial heterogeneity, and a long evolutionary history provide the basis for the formation of China's rich and diverse flora. Therefore, China is an important region for species diversity and an important center for species conservation, speciation, and evolution in the Northern Hemisphere (Axelrod, 1959; Ye et al., 2024). In recent decades, many systematic phylogeographical studies in China have focused on woody plants in the Tibetan Plateau and its adjacent areas (Himalayas and Hengduan Mountains; Li et al., 2013; Liu et al., 2013; Opgenoorth et al., 2010; Sun et al., 2014) and shrub species (Gao et al., 2015; Wang et al., 2009, 2010), most phylogeographical studies focus on genetic structure, genetic differentiation and the location of refuge during Quaternary glaciation, and few studies have studied the expansion and contraction routes of plants during post‐ice age (Dai et al., 2015; Li et al., 2023; Tian et al., 2015). Compared to long‐lived woody plants, herbs go through a much longer life cycle in a given amount of time and are therefore expected to respond much more quickly to environmental changes. Thus, herbaceous plants may provide a better opportunity to study the drivers of diversification and speciation (Comes & Kadereit, 1998; Xie et al., 2012). However, as far as we know, only a few herbs from the region, such as *Dysosma versipellis*, *Primula ovalifolia*, and *Oryza stavia*, have been studied to date (Guan et al., 2010; Liu et al., 2016; Qiu et al., 2009; Xie et al., 2012). In addition, phylogeography studies of plants distributed over a large range, across different climatic zones, and in multiple tropical biodiversity zones in China are still lacking.

*Pinellia ternata* (Thunb.) Breit is a perennial herb belonging to the genus *Pinellia* in Araceae, it is commonly used in Chinese medicine in clinical practice, growing in moist, warm, cool, loose sandy soil below 2500 m above sea level. It is endemic to East Asia and is found in China, Korea, and Japan. *P. ternata* is found in all provinces of China except Inner Mongolia, Qinghai, Xinjiang, and Tibet (Chen et al., 2020; Li et al., 2004). With the continuous expansion of the utilization range of *P. ternata*, the related research is also more active. The research mainly focuses on chemical composition, pharmacological action, and source of crude drugs (Guo et al., 2022; Lee et al., 2021; Noushahi et al., 2021). Studies at the molecular level started late, among which, molecular markers based on chloroplasts (Cui et al., 2022; Zhao & Li, 2016), ITS markers (Zhang, 2007), and SSR markers (Xu et al., 2024) have studied the relationship between genetic diversity and phylogeny of *P. ternata*, but the research basis at the molecular level is still quite weak, which is represented by insufficient sample size. *P. ternata*, as a widely distributed species with strong ecological adaptation, spans different climatic zones and many biodiversity research hotspots and is an ideal material for the study of herbal phylogeography. It is sensitive to climate change and environmental fluctuations, and its DNA base substitution rate is higher than that of woody plants, which can provide more information about historical evolution. The genetic information and evolutionary speed between cpDNA and nuclear DNA (nrDNA) are different, and microsatellite markers are co‐dominant markers, that can distinguish homozygous and heterozygous, detect multiple alleles, and have the advantages of rich polymorphism, simple operation, reliable results, and good repeatability (Yang et al., 2022). Therefore, differences in molecular markers may lead to different genetic structures (Cao et al., 2016; Hancock, 1999; Qi et al., 2012; Wolfe et al., 1987). Thus, multiple molecular markers can complement each other, which will contribute to a more objective understanding of the genetic structure of a species (Lowe et al., 2004).

Therefore, this study used chloroplast genes (*psb*K‐*psb*I and *trn*L‐F), nuclear genes (ITS and ETS), and SSR sequences for PCR amplification, sequencing, and analysis, with the main purpose of solving the following problems: (1) What are the historical dynamics of *P. ternata* populations? (2) What is the genetic structure and genetic differentiation of *P. ternat*a? (3) Are there dilatation‐contraction routes in *P. ternata* groups before and after glacial periods? If so, what is the route? (4) During the Quaternary glaciation, did the population in the area come from a common refuge or multiple sanctuaries, and where did its glaciation refuge reside?

## 2 MATERIALS AND METHODS

### 2.1 Sampling, experimental design, and DNA extraction

**FIGURE 1:**
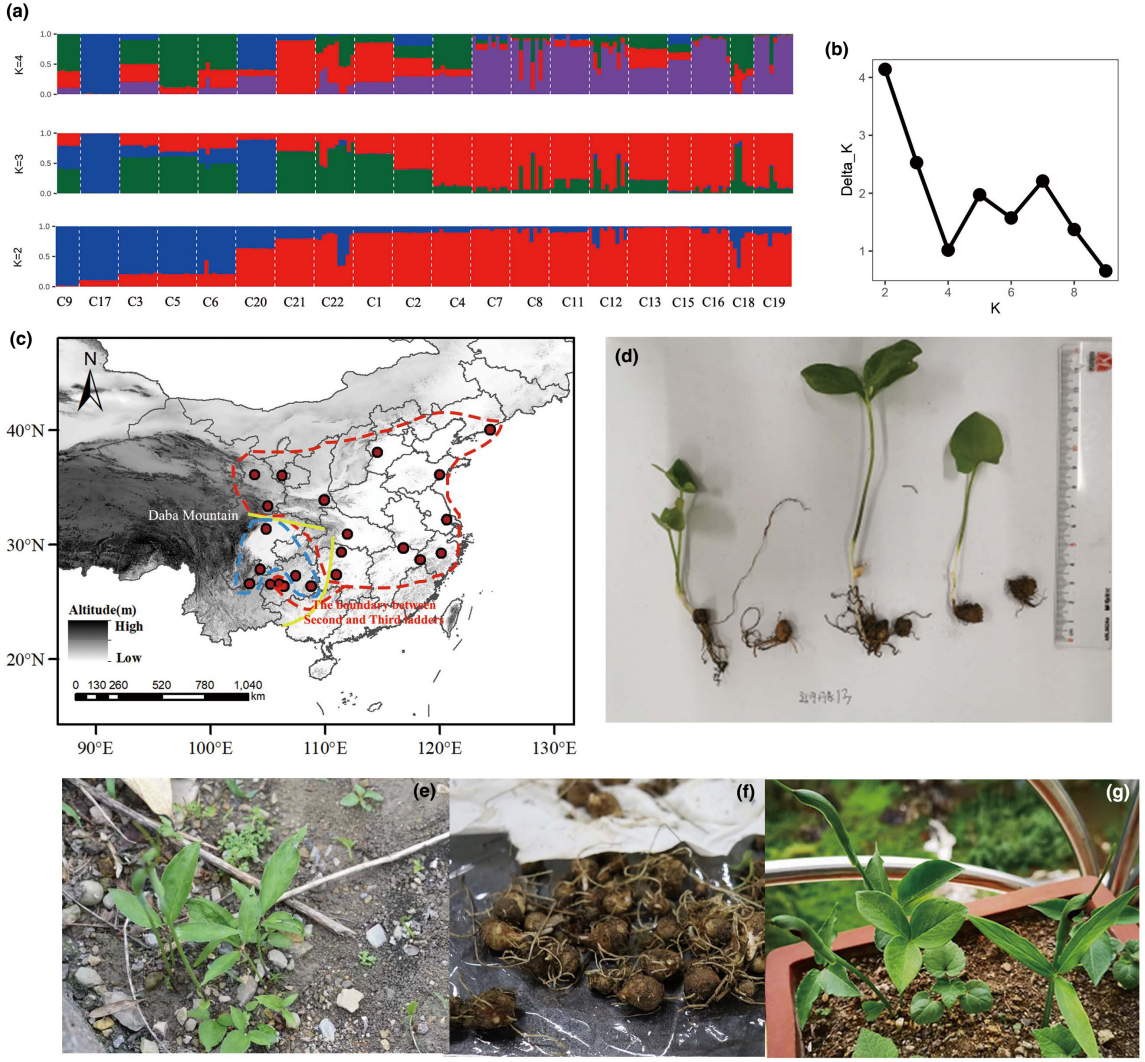
*Pinellia ternate* structure group and plant morphology. (a) Division of different groups when *K* = 2–4; (b) The rate at which the value of *K* changes from 1 to 10 corresponding to Delta *K*; (c) Geographical distribution map of population sampling points, which are divided into three groups according to SSR groups with corresponding colors, the two dots not circled in the figure represent population C10 and population C14. The two yellow lines represent the Daba Mountain and the boundary between second and third ladders of China respectively; (d–g) Leaf morphology and root morphology of *P. ternate*. The dashed line circled in blue is Group I (Southwest group), and the dashed line circled in red is Group II (Central and Eastern group).

For this study, more than 270 individuals from 22 populations of *P. ternata* were collected throughout the distribution range, with sample size per population ranging from 6 to 16 depending on the population size. The individuals collected were ≥ 5 m and each population is separated by more than 10 km apart to avoid sampling bias toward closely related individuals (see Table 1 for detailed information about the samples). Healthy and pest‐free fresh leaves were taken from each sample and quickly dried using a silica gel. The voucher specimens number for collecting samples were numbered from ZhaocBX1 ~ 22 and deposited in the plant herbarium of college of life science, Guizhou University. Sample sizes for *Machilus thunbergii* (individuals from 2 to 8), *Gentiana crassicaulis* (individuals from 3 to 15) and *Morella nana* (individuals from 2 to 12, our studies have been published.) were unbalanced in other phylogeographic studies, but it can cover the representative populations and geographical distributions (Fan et al., 2022; Ni et al., 2022; Wu et al., 2024). Nevertheless, the samples we collected covered the entire geographical distribution of the species and we attempted to collect as many populations and individual plants as possible in our study. Among them, there are 20 representative populations and 188 individuals used for SSR molecular marker selection (Table S1). Chloroplast DNA (cpDNA) and ribosomal DNA (nrDNA) segments of over 270 individuals from 22 natural populations were sequenced, population structure and genetic diversity were analyzed, and cpDNA sequence and nrDNA sequence results were used for phylogenetic analysis. Total genomic DNA was isolated from dry leaves by the modified CTAB method (Doyle, 1991). The *P. ternata* morphology can be seen in Figure 1. In SSR study, repeated amplification of C10 and C14 populations failed to obtain effective experimental data, so these two populations were abandoned in SSR study. In all subsequent processes involving SSR data analysis, 20 populations were used for research. However, to verify our results, we continued to study these two populations in cpDNA, ITS, and ETS sequences.

**TABLE 1:** Geographical distribution of sampled populations of *Pinellia ternata*.

Sample name	Sample size	Location	Longitude (E)	Latitude (N)	Altitude (M)
C1	14	Dandong, Liaoning	124.3541	40.01304	22
C2	15	Dangyang, Yichang, Hubei	111.9413	30.92048	59
C3	16	Liping, Qiandongnan, Guizhou	108.7476	26.39719	644
C4	15	Tianshui, Gansu	103.8709	36.07785	1085
C5	10	Zhaotong, Yunnan	104.3199	27.8321	897
C6	15	Zhaotong, Yunnan	103.3744	26.5735	2142
C7	15	Chizhou, Anhui	116.8293	29.6895	34
C8	15	Nantong, Jiangsu	120.6055	32.1733	10
C9	6	Kaiyang, Guizhou	107.4255	27.29947	1263
C10	12	Xingyi, Guizhou	105.2546	26.52304	1224
C11	14	Shaoyang, Hunan	110.9461	27.36621	302
C12	12	Changde, Hunan	111.4357	29.32264	68
C13	15	Yiwu, Zhejiang	120.1234	29.22179	91
C14	6	Guiyang, Guizhou	106.4177	26.38253	1109
C15	6	Shangluo, Shanxi	109.91	33.89973	702
C16	12	Shijiazhuang, Hebei	114.572	38.01232	75
C17	15	Mianyang, Sichuan	104.8536	31.38133	439
C18	6	Longnan, Gansu	104.9881	33.39363	898
C19	11	Shangyao, Jiangxi	118.2978	28.67076	26
C20	15	Qingdao, Shandon	119.9825	36.08664	16
C21	13	Guyuan, Ningxia	111.7865	32.92997	1955
C22	12	Bijie, Guizhou	106.0321	26.55288	1312

### 2.2 SSR marker amplification, cpDNA, and nrDNA sequencing

Two chloroplast gene fragments (*psb*K‐*psb*I and *trn*L‐F) and two pairs of nuclear gene fragments (nrDNA ITS and nrDNA ETS) were amplified and sequenced from more than 270 individuals. Primer design and PCR amplification were performed as described by Zheng et al. (2013). The sequencing reaction was performed at Bioengineering (Shanghai) Co., LTD., using the same primers as those used in the respective PCR. According to the results of cpDNA and nrDNA data analysis, four representative *P. ternata* populations were selected for transcriptomic sequencing, which were C4, C6, C8, and C13, in combination with the characteristics of different branches and distribution regions of *P. ternata*. Total RNA was extracted using Tiangen's RNAsimple total RNA extraction kit. The sequencing and partial data analysis were completed by Shanghai Ouyi Biomedical Technology Co., LTD. According to the transcriptome sequencing results, nine pairs of primers matching *P. ternata* SSR markers were finally screened.

### 2.3 Simulation of past species distributions using MaxEnt

A distribution record of *P. ternata* plants was downloaded from the Global Biodiversity Information Facility (GBIF). Based on the species distribution records and literature records recorded in this study, in order to prevent overfitting of the model caused by data duplication and spatial autocorrelation, the spatially overlapping data points within 10 km were eliminated (Dai et al., 2022), and 143 *P. ternata* distribution points were obtained, which better covered the distribution range of *P. ternata* in China. In ArcGIS 10.2, distribution points with distances less than 10 km are excluded. Nineteen bioclimatic factors for the current, Middle Holocene (about 6 kya), last glacial maximum, and future (2050) were downloaded from WorldClim (WorldClim). Bioclimatic variables corresponding to species distribution points were extracted and imported into MaxEnt v.3.4.1 together with species data for suitability prediction. Climate variables whose ecological contribution rate was greater than 1% in the model prediction results were retained (Shi et al., 2021). In order to eliminate the multicollinearity effect, we discarded the variable with the smaller biological contribution rate from Pearson's |*R*| ≥ 0.7 (Yusup & Sulayman, 2018), and finally selected 8 variables as climate predictors. The MaxEnt 3.4.1 software used 75% of the distributed data as a training set and the remaining 25% as a test set to model potentially suitable regions for past, present, and future climates of *P. ternata*.

### 2.4 Data analysis

Chromas software was used to view the base peak map of cpDNA and nrDNA sequenced sequences, and DNAstar SeqMan software (Burland, 1999) was used to arrange the arranged sequences para‐wise and splicate them in the positive direction, the software Bioedit 7.0.9.1 was used to calculate the GC content (Hall, 1999). After sequence correction with MEGA7.0 software, phylogenetic trees are built by PhyloSuite v1.1.15 (Zhang et al., 2020) after the formed data files are joined together. DnaSP v.6.0 (Librado & Rozas, 2009) software was used to calculate the number of haplotypes, haplotype diversity (Hd), and nucleotide diversity (π). PermutCpSSR‐2.0 software was used (Pons & Petit, 1996) to calculate the total genetic diversity (*H*_T_). The haplotype network map was constructed using NETWORK 10.20 software (Bandelt et al., 1999), and the haplotype geographic distribution map was drawn by ArcGIS 10.2 software. The software DnaSP v.6.0 was used for mismatch analysis and neutrality test of the cut sequences (Fu, 1997). Molecular analysis of variance (AMOVA) used Arlequin 3.5 software (Excoffier & Lischer, 2010) to calculate the degree of genetic variation within and among *P. ternata* populations and the coefficient of genetic differentiation (*F*_st_) among populations. PermutCpSSR‐2.0 software was used to calculate the population genetic differentiation coefficients (*G*_st_ and *N*_st_) of *P. ternata* (1000 random displacement test), so as to test whether there were obvious lineages and geographical structures among *P. ternata* populations. In addition, BEAST v 2.7.6 was used to analyze the dynamic change of population size over time using the Bayesian Skyline Plot method (BSP) (Bouckaert et al., 2019). We use the average base mutation rates (cpDNA: 2 × 10^−9^ substitutions/site/year; ITS: 7 × 10^−9^ substitutions/site/year) that Wolfe et al. (1987) previously estimated for the angiosperm cpDNA (1–3 × 10^−9^ substitutions/site/year) and ITS (5.8–8.1 × 10^−9^ substitutions/site/year), alongside a population coalescent Bayesian Skyride model for the prior tree and a relaxed molecular clock (uncorrelated lognormal), as well as previous tree population condensation Bayes Skyride model and relaxation molecular clock. Use a random initial tree, linear model, and set the MCMC chain length to ESS ≥200 (100,000,000 chains for cpDNA and ITS datasets). We performed three independent analyses and combined them with TRACER 1.5. ETS fragments of nrDNA can provide a large amount of species information but are not suitable for population size dynamics and differentiation over time analysis due to the rapid base replacement rate (Shahzad et al., 2017). Therefore, we did not use these datasets for the analysis of Bayesian skylines in our study.

The sequencing and partial data analysis of transcriptional library were completed by Shanghai Ouyi Biomedical Technology Co., LTD. Trimmomatic software (Bolger et al., 2014) was used for quality control and removal of the sequenced data, and Trinity 2.4 software (Grabherr et al., 2011) spliced the Transcript sequence and selected the longest one as Unigene according to the sequence similarity and length. Then the Unigene sequence was compared with the above protein libraries (evalue<1e‐5), and the coding region was translated into amino acid sequence. Finally, for Unigene that did not match the above protein libraries, ESTScan 3.0.3 software was used to predict its coding region, and the nucleic acid sequence (sequence direction 5′→3′) and amino acid sequence of the coding region were obtained (Iseli et al., 1999). The software MISA was used to predict SSR and Primer3 software was used to design SSR primers. According to the SSR study, GenAlex 6.5 software (Peakall & Smouse, 2012) was used to calculate the expected heterozygosity (*He*) and observed heterozygosity (*Ho*) effective number of allele (*Ne*), observed allele number (*Na*), Shannon information index (*I*) and other genetic parameters of 20 *P. ternata* populations. The software CERVUS 3.0 was used to calculate the polymorphic information content (PIC) of each SSR site (Kalinowski et al., 2007). GenAlex 6.5 software was used to complete Nei's genetic distance detection, Mantel test, and principal coordinate analysis (PCoA), and MEGA 7.0 software was used to construct adjacent tree (NJ) according to the results of Nei's genetic distance. The genetic STRUCTURE of 20 *P. ternata* populations was analyzed using STRUCTURE 2.3.4 software (Falush et al., 2003). Before calculation, set the population Number *K* value to 1–10, Length of burn‐in period = 10,000, Number of MCMC Reps after Burnin = 100,000, and repeat the calculation 20 times. To upload the result, Structure Harvester Web v0.6.94 Harvester (http://taylor0.biology.ucla.edu/structure) (Earl & Vonholdt, 2012) is analyzed, the magnitude of the rate of change of L (*K*) between successive *K* values is obtained, and the most probable *K* value is determined by the *K* between successive *K* values. Finally, the cluster diagram of STRUCTURE is obtained (Evanno et al., 2005). Bottleneck 1.2.02 (Cornuet & Luikart, 1996) is based on infinite allele model (IAM). Twenty populations of *P. ternata* were detected by stepwise mutation model (SMM) and two‐phased model of mutation (TPM) to determine whether multiple loci of *P. ternata* population showed excessive heterozygosity and experienced bottleneck effect. Parameters are set as follows: Variance for TPM = 30, Proportion of SMM in TPM = 70%, Iterations = 1000. Monmonier's maximum‐difference algorithm was calculated using Barrier v2.2 (Manni et al., 2004), which was used to identify biogeographic boundaries or regions showing the greatest genetic discontinuity between populations. We used cpDNA and SSR data to select 22 and 20 populations respectively for Barrier analysis.

## 3 RESULTS

### 3.1 Genetic diversity and genetic structure based on SSR sequences

Combined with transcriptomic sequencing results, the amplification and genetic diversity of 188 *P. ternata* individuals from 20 different populations were analyzed using nine pairs of SSR primers. The mean observed number of alleles (*Na*), the mean effective number of alleles (*Ne*), the mean Shannon information index (*I*), the mean observed heterozygosity (*Ho*), the mean expected heterozygosity (*He*), and the mean polymorphism information content (PIC) were 2.210, 1.880, 0.700, 0.745, 0.432, and 0.731, respectively (Table 2). In summary, primer SSR8 revealed the highest level of genetic diversity, while primer SSR6 revealed a lower level.

**TABLE 2:** Genetic diversity of SSR primers.

Primer	*Na*	*Ne*	*I*	*Ho*	*He*	PIC
SSR1	2.05	1.724	0.656	0.7	0.406	0.873
SSR2	2.5	2.295	0.804	0.95	0.523	0.511
SSR3	3.35	2.643	1.039	0.95	0.6	0.732
SSR4	2.55	2.205	0.816	1	0.538	0.671
SSR5	2.4	2.042	0.765	0.75	0.456	0.778
SSR6	0.9	0.9	0.312	0.45	0.225	0.475
SSR7	2.9	2.478	0.928	1	0.576	0.86
SSR8	3.5	2.867	1.051	1	0.61	0.832
SSR9	1.95	1.641	0.634	0.65	0.387	0.844
Mean	2.21	1.88	0.7	0.745	0.432	0.731

Abbreviations: *He*, expected heterozygosity; *Ho*, observed heterozygosity; *I*, Shannon information index; *Na*, observed allele number; *Ne*, effective number of allele; PIC, polymorphic information content; Primer, primer name.

The analysis of population molecular variance (AMOVA) based on SSR markers showed that the genetic variation of *P. ternata* mainly occurred within the population, accounting for 86% of the total variation (Table S8). Mantel test for 20 populations of *P. ternata* showed no significant association between geographic distance and Nei's genetic distance (*r* = .0608, *p* = .03 < .05, Figure S1), indicating that geographic distance was not the main factor leading to genetic differentiation of *P. ternata*.

Genetic Structure analysis based on structure shows that *K* = 2, the curve of Delta *K* changing with *K* value corresponds to the largest value (Figure 1b). It is most reasonable to divide the 20 populations of *P. ternata* into two groups, namely the Southwest group (group I), the Central and eastern group (group II), group I and group II were separated roughly by Daba Mountain and the boundary between second and third ladders of China (Figure 1c). Some individuals of 20 *P. ternata* populations were mixed to different degrees, indicating that there was a certain degree of gene exchange among the populations. The neighboring tree of 20 *P. ternat*a populations was constructed based on Nei's genetic distance, there is generally consistency between the results of systematic clustering and the results of structure analysis (Figure S2). According to the results of Barrier analysis, there was a tendency of genetic boundary between the first and second groups, and a certain degree of mixing occurred between different populations (Figure S3).

### 3.2 Genetic diversity and genetic structure based on cpDNA and nrDNA sequences

**FIGURE 2:**
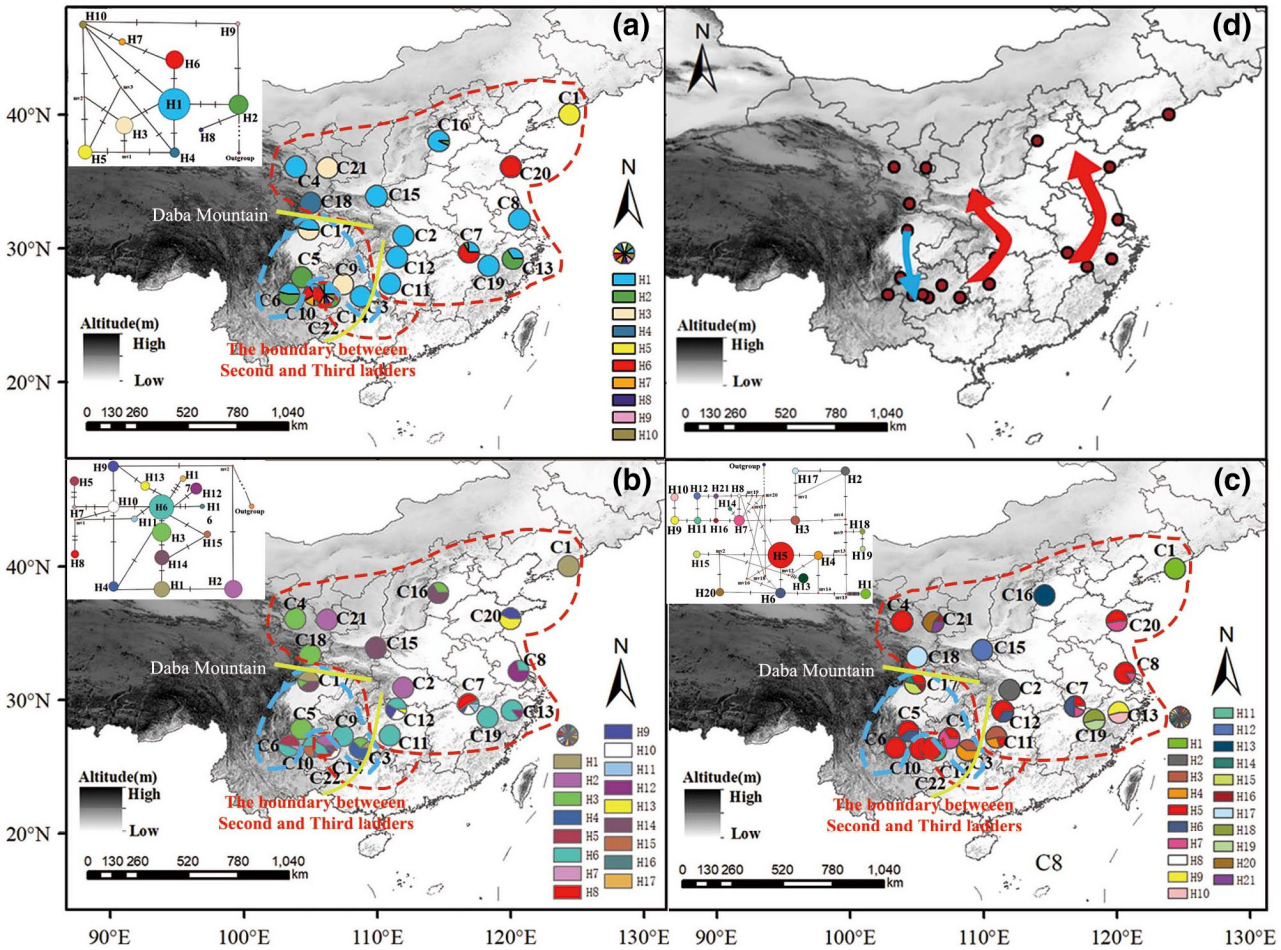
Haplotype geographic distribution and expansion road. (a) Geographic distribution of haplotype based on cpDNA, the pie chart shows the frequency of haplotypes in each population, and the upper left corner is a haplotype network based on cpDNA; (b) Geographic distribution of haplotype based on nrDNA ITS, the pie chart shows the frequency of haplotypes in each population, and the upper left corner is a haplotype network based on cpDNA; (c) Geographic distribution of haplotype based on nrDNA ETS, the pie chart shows the frequency of haplotypes in each population, and the upper left corner is a haplotype network based on cpDNA; (d) *Pinellia ternata* extension path. The blue arrow indicates the first group of extension paths, and the red arrow indicates the second group of extension paths. The dashed line circled in blue is the first group (southwest Group), and the dashed line circled in red is the second group (Central and eastern group). The two yellow lines represent the Daba Mountain and the boundary between second and third ladders of China respectively.

According to SSR analysis results, cpDNA, ITS, and ETS data of *P. ternate* were divided into two groups for analysis. The cpDNA union sequence (*psb*K‐*psb*I + *trn*L‐F), nrDNA (ITS), and nrDNA (ETS) of over 270 individuals were sequenced successfully, and the corrected sequence lengths were 1312, 743, and 570 bp, respectively. There were 7, 16, and 18 variation sites, and the G + C content was 31.20%, 65.68%, and 65.79%, respectively. A total of 10 cpDNA haplotypes (H1–H10), 17 ITS haplotypes (H1–H17), and 21 ETS haplotypes (H1‐H21) were identified (Figure 2). Three networks of *P. ternate* were constructed based on the cpDNA, ITS, and ETS haplotypes, with *Pinellia pedatisecta* as an outgroup (Figure 2).

The results showed that cpDNA haplotype H1 was the most common, occurring in 148 individuals and the most widely distributed, occurring in 16 populations. According to coalescent theory, the most ancient haplotypes should be located at the interior nodes of a haplotype network, whereas the most recent haplotypes should be at the tips (Posada & Crandall, 2001). According to the haplotype network analysis results, other haplotypes were derived from H1 as the center and cpDNA haplotype H1 was presumed to be an ancestral haplotype (Table S2). ITS haplotype H6 appeared in 76 individuals with the highest frequency and widest distribution, and other haplotypes were derived from H6. Therefore, ITS haplotype H6 was presumed to be an ancestral haplotype. The haplotype H5 of ETS showed the highest frequency and widest distribution, with 96 individuals distributed in 12 populations (Table S4). According to the haplotype network analysis results, all haplotypes in the haplotype network diagram were branches of haplotypes derived from H5, and haplotype H5 was located in the internal nodes of the network adjacent the outgroup, so it could be inferred that the haplotype H5 of ETS was an ancestral haplotype. In addition, multiple chloroplast haplotypes were found in five populations, multiple ITS haplotypes in 11 populations, and multiple ETS haplotypes in 12 populations. The total haplotype diversity Hd of cpDNA, nrDNA (ITS), and nrDNA (ETS) was 0.665, 0.8683, and 0.847, respectively, and the total nucleotide diversity π was 0.97 × 10^−3^, 3.03 × 10^−3^, and 7.60 × 10^−3^, respectively. The haplotype diversity of C7, C10, C13, and C22 populations was higher than that of other populations (Figure 2a–c; Tables S2–S4).

The inter‐population genetic differentiation coefficients (*G*_ST_) were 0.042, 0.620, and 0.614, and the *N*_ST_ were 0.187, 0.729, and 0.878, respectively. The inter‐population genetic differentiation coefficients were all *N*_ST_ > *G*_ST_ (.01 < *p* < .05), indicating that different haplotypes with similar genetic relationships appeared in the same population at the population level. *P. ternata* has an obvious molecular pedigree geographical structure.

The results of molecular variance analysis (AMOVA) of cpDNA, ITS, and ETS fragments of all *P. ternata* populations showed that the inter‐population genetic variation was 77.23%, 72.12%, and 86.65%, respectively, and the intra‐population genetic variation was 22.77%, 27.88%, and 13.35%, respectively (Tables S5–S7). The genetic differentiation coefficients (*F*_ST_) were 0.772, 0.720, and 0.866, respectively, all greater than 0.25 (*p* < .001), meaning that the degree of genetic differentiation among *P. ternata* populations had reached a significant level and there was obvious isolation. Assuming *psb*K‐*psb*I + *trn*L‐F combined fragment, ITS fragment, and ETS fragment variation were in drift‐migration equilibrium, the average gene flow values (*N*m) among *P. ternata* populations at the species level were estimated based on *F*_ST_ values to be 0.02, 0.09, and 0.04, respectively. These results indicate that the gene flow between *P. ternata* populations is small and the gene exchange between populations is not frequent.

### 3.3 Inference of demographic history

**FIGURE 3:**
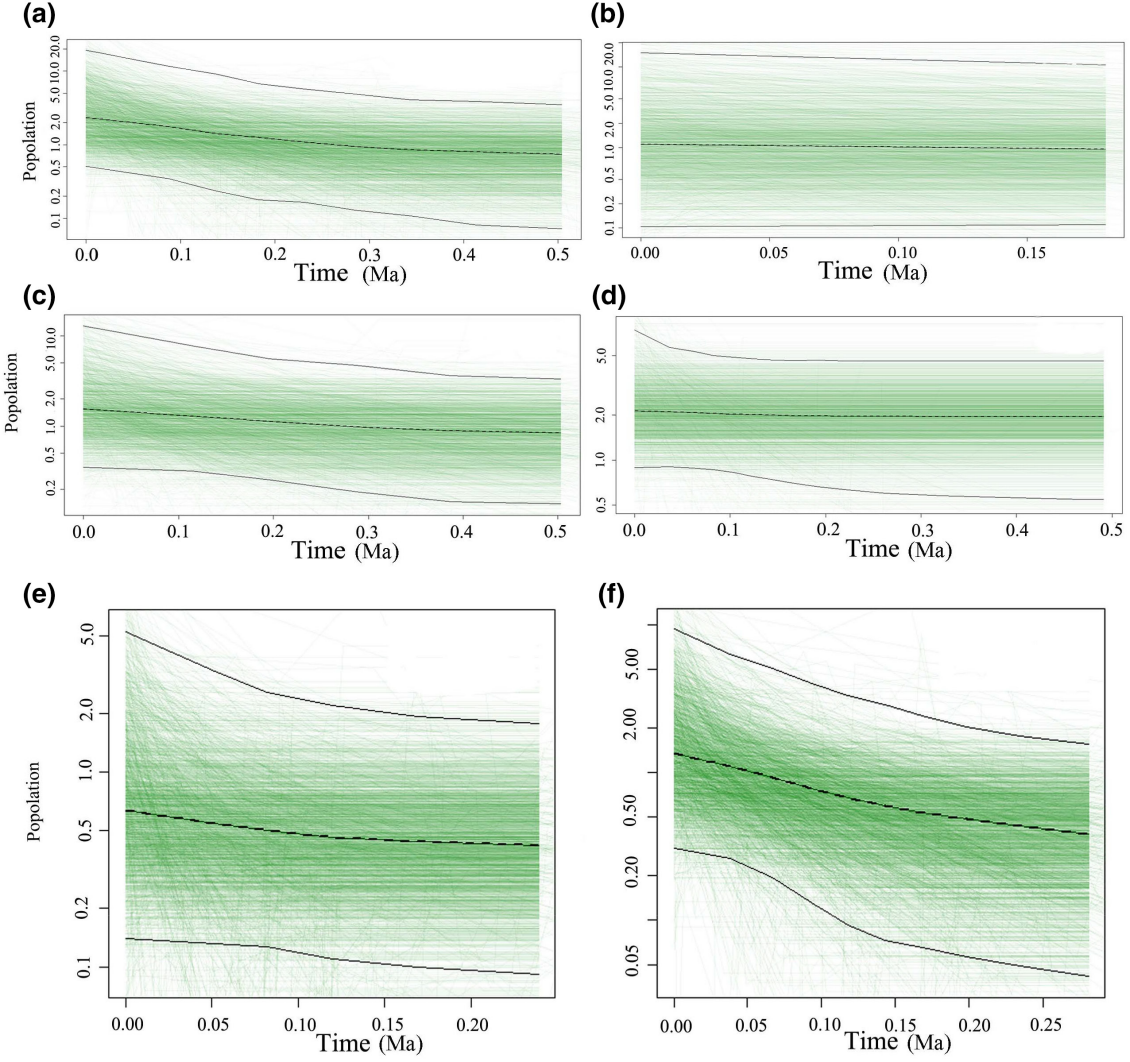
Bayesian skyline plot for *Pinellia ternate* populations. (a) Bayesian skyline map of *P. ternate* populations (all populations) based on cpDNA data; (b) Bayesian skyline map of *P. ternate* population based on cpDNA data (Group I population); (c) Bayesian skyline map of *P. ternate* population based on cpDNA data (Group II population); (d) Bayesian skyline map of *P. ternate* population based on ITS data (all populations); (e) Bayesian skyline map of *P. ternate* population based on ITS data (Group I population); ITS Group 1. (f) Bayesian skyline map of *P. ternate* population based on ITS data (Group II population). The black dashed line indicates the median values of effective population size (Ne). The upper and lower black lines represent the boundaries of the highest 95% posterior density (HPD) interval of Ne.

The neutral test and mismatch analysis of cpDNA, ETS, and ITS of *P. ternata* showed that the conservative statistical values of Tajima's *D* were 0.25544 (*p* > .10), 0.44116 (*p* >.10), and −0.32485 (*p* > .10), respectively. The values of Fu and Li's D* were 0.13295 (*p* >.10), 1.83383 (*p* > .10), and 0.36671 (*p* > .10). Fu and Li's F* values were 0.21087 (*p* > .10), 1.52593 (*p* > .10), and 0.11497 (*p* > .10). Fu's Fs values were 2.849, −0.894 (*p* > .10), and −3.053. The mismatch distribution of cpDNA and ETS actually showed multiple peaks in the expansion curve, which was contrary to the expected value and the population expansion model, and no population expansion signal was detected in the neutral test results, indicating that the population history was stable and *P. ternata* did not experience rapid expansion events (Figures S4 and S5). The mismatch distribution of ITS actually shows a unimodal distribution on the expansion curve, which is the same as the expected value and the population expansion model, and the population expansion signal is detected in the neutral test results, indicating that *P. ternata* experienced a rapid expansion event (Figure S6). Both the observed value curve and the expected value curve of ITS mismatch analysis showed a downward trend and were unipeak distribution curves, indicating that *P. ternata* population experienced an expansion event recently. The bottleneck effect of the population was detected based on the three models, and no bottleneck effect was detected (Table S9). However, the results of BSP analysis showed that the *P. ternata* experienced an expansion event. The results of BSP analysis using cpDNA data showed that the median effective population size of *P. ternata* increased significantly from about 0.2 Ma (Figure 3a), and the results of BSP analysis using ITS data showed that the median effective population size of *P. ternata* increased significantly from about 0.15 Ma (Figure 3d). This indicates that the overall *P. ternata* began to expand from about 0.15 to 0.2 Ma. In addition, BSP analysis of *P. ternata* population grouping also verified that *P. ternata* had expanded. ITS data showed that group I began to expand at about 0.15 Ma (Figure 3e), while cpDNA data showed that Group I did not expand (Figure 3b). The cpDNA data showed that group II began to expand at about 0.2 Ma (Figure 3c), and ITS data showed that group II began to expand at about 0.15 Ma (Figure 3f). Therefore, the results of BSP analysis indicated that the *P. ternata* underwent an expansion event.

### 3.4 Analysis of suitable establishment areas for *P. ternata*

**FIGURE 4:**
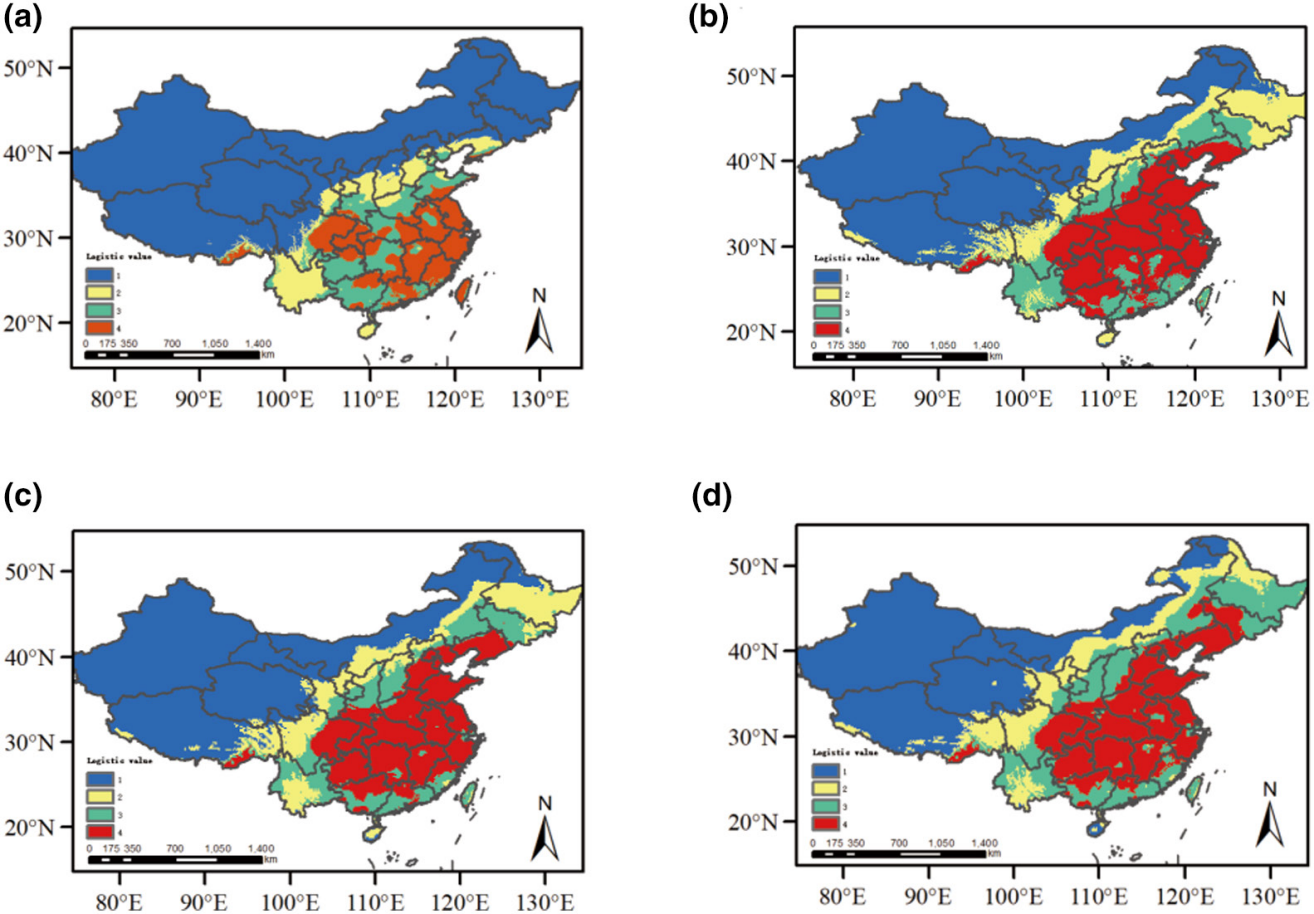
Simulation of the distribution area of *Pinellia ternata* in different climatic conditions in China. (a) Last Glacial Maximum (LGM); (b) Mid‐Holocene; (c) Current; (d) Future in 2070. In the figure, red represents highly suitable areas, green represents moderately suitable areas, yellow represents low suitable areas, and blue represents unsuitable areas.

MaxEnt software was used to predict the potential distribution of *P. ternata* in China. The predicted value was (AUC = 0.981), which could be used to simulate the potential distribution area of *P. ternata*. The results showed that: in LGM, the global climate cooled sharply, the suitable areas of *P. ternata* decreased significantly, and the high‐suitable areas contracted into the southwest, central, and eastern parts, indicating that the Quaternary glaciation refuge of *P. ternata* may be located in these places (Figure 4a). Therefore, the general trend of *P. ternata* is to move south. The Middle Holocene climate was warm and humid, similar to the modern climate, with a marked expansion of the suitable *P. ternata* area during the Middle Holocene (Figure 4b). From the middle Holocene to the future, the distribution of *P. ternata* will expand, and the overall suitable area of *P. ternata* will reach its maximum in the future, but the current area of high suitable area has reached its maximum and will shrink slightly in the future (Figure 4c,d).

## 4 DISCUSSION

### 4.1 Population genetic diversity and genetic structure

In this study, genetic variation estimated using cpDNA and ITS sequence data was higher than the average genetic diversity of angiosperms (Petit et al., 2005; Taberlet et al., 1991). These results were further supported by SSR molecular markers, indicating a high genetic diversity estimated in this study. This may be due to the accumulation of rich chloroplast genetic variation during the long evolutionary history and generational alternation. The haplotype diversity and total genetic diversity of chloroplast genes (cpDNA: *H*_T_ = 0.892) were higher than those of nuclear genes (ITS: *H*_T_ = 0.816; ETS: *H*_T_ = 0.846). The estimated genetic diversity of chloroplast genes was higher than that of some herbs, such as *Notopterygium forrestii* (*H*_T_ = 0.623; Shahzad et al., 2017) and *Panzerina lanata* (*H*_T_ = 0.577; Zhao et al., 2019). The genetic diversity of a population is caused by many factors, such as the geographical distribution of the species, biological characteristics, population size, and reproductive system (Petit et al., 2005). The high level of genetic diversity of *P. ternata* may be related to its breeding system, geographical distribution, and environmental factors (Wang et al., 2012). First of all, *P. ternata* contains tuber propagation, plant bud propagation, and seed propagation. Meanwhile, *P. ternata* also has a complex gene pool (Li et al., 2023), the sexual reproduction of *P. ternata* can provide genetic information, improve genetic variation, and provide a rich material basis for gene mutations, resulting in a high genetic diversity of *P. ternata* population. Second, *P. ternata* is widely distributed in all provinces and regions except Inner Mongolia, Qinghai, Xinjiang, and Tibet. Previous studies have shown that the geographical distribution of species is closely related to genetic diversity (Hamrick & Sherman‐Broyles, 1992), so the wide geographical distribution may also be the reason for the high genetic diversity of *P. ternata*. Finally, *P. ternata* can grow in grassy slopes, wastelands, cornfields, fields, or under sparse forests below 2500 m above sea level, and the living environment is also one of the reasons for the high level of genetic diversity of *P. ternata*. In addition, if the bottleneck effect occurs in the population, genetic drift will change the allele frequency to a large extent, resulting in changes in the genetic structure and diversity level of the restored species population, resulting in a decrease in the genetic diversity level of the population (Li et al., 2023). In this study, the bottleneck effect of *P. ternata* population was detected, and the results showed that all populations were not affected by this effect, so this is also a reason for the high level of genetic diversity in *P. ternata*.

Complex terrain generated by tectonic movements and Quaternary glacial–interglacial cycles may also lead to reduced gene flow and intraspecific allogenic differentiation (Liu et al., 2018). In addition, due to geographical barriers (deserts, mountains, etc.), species separation, seed spread limitation, and less genetic exchange between populations lead to the separation and differentiation of genetic lineages. The genetic structure of species populations is affected by many factors such as geological and historical climate changes, topographic conditions, habitat heterogeneity, and the degree of seed gene exchange (Liu et al., 2021). In this study, fragment analysis of cpDNA, ITS, and ETS showed that the *N*_ST_ value was higher than the *G*_ST_ value, and the *p*‐value was lower than 0.05. *F*_ST_ values were all greater than .7, *p* < .001. The results of cpDNA, ITS, and ETS markers indicate that the main source of variation in *P. ternata* is between populations, whereas SSR indicates that the main source of variation is within populations, a contrast that usually indicates higher genetic diversity within species. In fact, the use of SSR markers tends to show more genetic variation within populations than between populations, such as *Iris loczyi* (Zhang et al., 2021), *Pseudotaxus chienii* (Li et al., 2024), *Acer pentaphyllum* Diels (Hao et al., 2019), *Camellia luteoflora* (Wang, 2020), the opposite results of molecular variation in the present study may have multiple reasons. First, the fruits of *P. ternata* are not favored by most animals, so the seed dispersal ability is very limited, and most of the seeds fall near the parent plant. Second, due to habitat fragmentation and human activities, gene exchange between *P. ternata* populations is more limited. Finally, *P. ternata* has undergone a complex evolutionary process during its long history, and the effects of geographic isolation or ecological isolation during long‐term evolution may also have contributed to the fact that genetic variation in *P. ternata* mainly originated within populations (Li et al., 2022). However, the results of cpDNA, ITS, and ETS markers in this study indicated that the genetic variation among *P. ternata* populations was greater than that within populations. Therefore, we concluded that the genetic variation of *P. ternata* mainly originated from inter‐population.

When the average gene flow *Nm* with *F*_ST_ value at the species level is less than 1, the gene flow is insufficient to resist population differentiation caused by genetic drift within the population. The main reason for the low gene flow of lianas may be related to breeding methods because the pearl buds, tubers, and fruits of lianas are relatively close to the mother plant and lack effective transmission methods, so they are poor pioneer species. Low level of gene flow may lead to population adaptation to local ecological environment, thus accelerating genetic differentiation among populations, and genetic drift may be the main factor affecting population genetic structure (Balloux & Lugon‐Moulin, 2002; Sun & Wong, 2001). Apart from reproductive mode and genetic drift, geographical isolation, ecological environment, and population history may also be the causes of genetic differentiation of *P. ternata*.

Our results showed that the *N*_ST_ was significantly greater than the *G*_ST_, suggesting a clear genealogical geographic structure of *P. ternata*. According to the results of structural analysis, 22 populations were divided into two groups according to the optimal **K** value. Geographic structure is common in plants with a continuous distribution, which is usually due to distance isolation or environmental isolation (Correa Ribeiro et al., 2016; Fiorini et al., 2020). In terms of geographic distribution, all populations are roughly separated by the Daba Mountains in the northern part of the Sichuan Basin and the boundary between the second and third ladders in China. The group I (Southwest group) is roughly located in the Sichuan Basin and the southwestern part of China, and the group II (Central and eastern group) is located in the northern part of the Sichuan Basin and the third ladders region of China. The role of the Daba Mountains and the boundary between the second and third ladders as geographic boundaries in plant genetic structure has been verified in previous studies (Kou et al., 2016; Zhan et al., 2009). In our study, the grouping of population C22 in the structure analysis is not entirely consistent with its geographic location. The STRUCTURE grouping results show that there is mixing between individuals from different populations, and it is particularly noteworthy that when *K* equals 2, there is significant mixing between individuals from C20 and C22. In addition, Barrier analysis also showed a tendency of mixing among different populations, such as C3 and C9. This result may be due to the fact that *P. ternata* is medicinal plants, which have high medicinal value, will be cultivated and introduced by human beings, which, after a long period of time, interferes with the original genetic structure, causing a few individuals in other populations to cross with some of the individuals. In addition, climatic oscillations have altered the distribution range of *P. ternata*, allowing mixing between different populations. This inconsistency in grouping results and geographical location is also seen in some species, such as *Allium sativum* (Li et al., 2021), *Mango cultivars* (A A Hussein et al., 2023), and *Fagus hayatae* (Ying et al., 2016).

There may be the following reasons for the formation of the current distribution pattern of *P. ternata*. Firstly, from populations C3, C5, C6, C9, and C17, it can be seen that the dividing line between the group I (Southwest group) and the group II (Central and eastern group) coincides with the Daba Mountains and the mountains south of the boundary between the second and third ladders. Therefore, the mountains on the boundary between the Daba Mountains and the boundary between the second and third ladders may act as diffusion barriers, preventing gene exchange between different populations on both sides. Mountains, as a geographical barrier, often hinder the gene exchange of species. Long‐term geographical isolation may shape a new genetic pattern of species, which has been verified in many studies, such as *Rhodiola*, *Himalrandia lichiangensis*, *Prunus pseudocerasus* (Chen et al., 2023; Qiao et al., 2022; You et al., 2022). Second, mountain orogeny increased topographic heterogeneity, brought about great changes in topography, watershed and climate, formed new habitat types, promoted speciation, and influenced the phylogeographic pattern of species (Hughes & Atchison, 2015; Liu et al., 2023; Zheng et al., 2021). Finally, climate oscillations often cause the range of many plants to contract or expand, and the glacial–interglacial cycles of the Quaternary have repeatedly altered the distribution of organisms, creating fluctuating population dynamics (Matías et al., 2017).

High haplotype and nucleotide diversity were characteristic of glacial refuges during Quaternary glaciation (Gong et al., 2008), and these stable and diverse environments were conducive to the maintenance of species richness. The cpDNA and nrDNA data analysis showed that populations C17 were found in northern Sichuan Basin (Southwest China), C7, C13, and C19, Jiangxi Province and Zhejiang Province in the downstream Yangtez river region (Eastern China). The populations of *P. ternata* in these regions have high genetic diversity, including ancient haplotypes and private haplotypes. The Sichuan Basin preserves a large amount of species diversity, and this region has been verified in many studies as a typical Quaternary glaciation refuge. For example, *Fagus hayatae* (Ying et al., 2016), *Osmanthus serrulatus* (Wang et al., 2024), and *Phoebe zhennan* (Xiao et al., 2020). Previous phylogeographical studies have also shown that during the Quaternary glaciation, southwest China and eastern China also proved to be protected areas for other species, such as *Ginkgo biloba*, *Shaniodendron subaequale*, *Chimonobambusa utilis*, *Cathaya argyphylla* (Gong et al., 2008; Lai et al., 2023; Liu et al., 2022; Wang & Ge, 2006). Therefore, it is speculated that there may be two glacial refuges in the Quaternary glacial period, which are located in the Sichuan Basin (Southwest China) and the downstream Yangtez river region (Eastern China).

### 4.2 Demographics and distribution dynamic

East Asia was not directly affected by the widespread emergence of ice sheets during the Quaternary period, but climatic fluctuations in the region, especially since the LGM period, have played an important role in shaping the distribution and spatial genetic patterns of existing species (Huang et al., 2024; Liu, 1988; Qiu et al., 2011). Based on historical demographic observations, cpDNA and ETS mismatch analysis and neutral test results did not detect population expansion signals, however, ITS mismatch analysis results indicate that *P. ternata* experienced a recent expansion event. In addition, BSP analyses based on cpDNA data indicated that the expansion of *P. ternata* began to occur around 0.2 Ma, and BSP analyses based on ITS data indicated that the expansion of *P. ternata* began to occur around 0.15 Ma, the results of the BSP analyses indicated that *P. ternata* experienced expansion events both overall and in subgroups. Therefore, the results of BSP analysis indicated that *P. ternata* had experienced an expansion event. Niche simulation results showed that during the LGM period, *P. ternata* contracted and migrated southward, and the high‐habitat region contracted to southwest China (Chongqing Municipality, Northern Sichuan basin, and northern Guizhou Province) and eastern China (Jiangxi, Anhui, and Zhejiang provinces). The general view is that species tend to shrink their range in refuges during glacial expansion and expand as the climate warms (Weng et al., 2023). In the Mid‐Holocene period, the global climate was warm and humid, which contributed to the large area expansion of *P. ternata*, and the future climate will continue to warm from the current basis, which will contribute to the continued expansion of *P. ternata*. This finding is consistent with a study in the mountains of Southwest China (Liang et al., 2018). Consistent with this, the study suggests that most species are expanding their range in response to climate warming, supporting the hypothesis of a large expansion in the Mid‐Holocene. In addition, haplotype distribution patterns can also verify plant expansion. For example, the study on *Tapiscia sinensis* found the presence of haplotype H6 in Wuyi Mountain and Dabie Mountain, and the presence of haplotype H11 in Luoxiao Mountain. This suggests that local range extension of *Tapiscia sinensis* occurs (Zhang, Li, Fritsch, et al., 2015; Zhang, Li, Liu, et al., 2015). Therefore, according to the results of this study, cpDNA haplotype H1 is widely distributed throughout our study area, including southwest China, central and eastern China, and northern China, ITS haplotype H6 is widely distributed in southern China, and ETS haplotype H5 is widely distributed in southwest and eastern China. From the distribution area of the dominant haplotype of these three sets of data, *P. ternata* is likely to have expanded.

The comprehensive analysis showed that a large area of *P. ternata* decreased in the LGM period due to global climate cooling, and the refuge of *P. ternata* decreased in the Quaternary glaciation, while there was a certain extent of expansion in the Mid‐Holocene period due to the temperature rotation. Glacial and interglacial cycles have resulted in several possible expansions and contractions of *P. ternata* and contacts between different shelters. This dynamic pattern fits with a general pattern of organisms responding to climate change, as species experience southward retreat and northward settlement during glacial/interglacial periods. Therefore, combining the results of mismatch analysis, BSP analysis, and niche simulation, we believe that *P. ternata* experienced an expansion event.

### 4.3 Repeated range expansions during inter/postglacial periods

Some haplotypes have evolved from ancient haplotypes, and the variation of the haplotype diversity in *P. ternata* reflects the expansion and contraction routes during the glacial and postglacial periods. Our study shows that there are three expansion‐contraction routes. During the Quaternary glaciation, the temperature dropped sharply in China, and *P. ternata* migrated from south to north, producing at least two ice age refuges in the Sichuan Basin (Southwest China) and the downstream Yangtez river region (Eastern China), preserving the ancient haplotype. The subsequent interglacial/postglacial period caused the temperature to rotate, and *P. ternata* began to expand producing three expansion routes, and this conclusion was also verified in the simulation results of suitable areas. These results are consistent with some previous studies (Li et al., 2012; Qi et al., 2012; Sun et al., 2014) highlighted the importance of distribution area expansion in shaping the geographic structure of plant species in China, providing new insights into the complex evolutionary history of plants in the region. In fact, in previous studies, the routes of plant expansion and contraction in China have been severely underestimated. Most studies have been limited to the phylogeographic structure of plants and the exploration of glacial refuges, but have failed to identify postglacial expansion routes (e.g., Liu et al., 2013; Qiao et al., 2022; Sun et al., 2014; Zhao et al., 2019). Among the widely distributed plants in China, multiple isolated refuges are common. However, the same haplotype or closely related haplotypes are often shared between multiple isolated shelters, implying repeated contact between shelters (e.g., H8 in Qiu et al., 2009; H1 in Shi et al., 2014). This phenomenon of repeated shelter contact is also common in European species (Park & Donoghue, 2019). The star spread of haplotype network maps around only one dominant haplotype indicates that the northern haplotypes arose from postglacial expansion from south to north. However, our haplotype network maps do not fully conform to this star network map. Therefore, we speculate that *P. ternata* produced partial autochthonous refuge during the glacial period, and during postglacial diffusion, the northern haplotypes were not fully dispersed. Repeated contact between *P. ternata* in autochthonous refugia and southern expanding populations eventually resulted in the current haplotype diversity and distribution pattern, and similar findings have been confirmed in *Ostryopsis davidiana* (Tian et al., 2009).

ETS and ITS data reflect the presence of some private haplotypes in different regions of *P. ternata* distribution range, for example, ETS haplotypes H9 and H19 in southern East China, H4 in Southwest China, ITS haplotypes H11 and H12 in Eastern China, and H5 and H15 in Southwest China. This suggests that *P. ternata* may have survived in some remote refuges during the Quaternary glaciation. However, cpDNA, ETS, and ITS data all found haplotypes shared in different glacial refuges and produced different derived haplotypes (cpDNA haplotype H1, ETS haplotype H5, and ITS haplotype H3). This phenomenon suggests that a late‐glacial expansion event carried haplotypes H1, H5, and H3 into different distribution regions of *P. ternata*, and that many derived haplotypes were produced in these isolated locations. Then, with the advent of the Holocene, these haplotypes participated in local expansion. We speculate that there are three expansion routes of *P. ternata*. It is worth mentioning that the first expansion route, the climate of Sichuan Basin in southwest China during the Quaternary glacial period, is more suitable for serving as a Quaternary glaciation refuge than that of Yunnan and Guizhou. This region preserves a large amount of species diversity, and the Sichuan Basin has been verified in many studies as a typical Quaternary glaciation refuge, such as *Fagus hayatae* (Ying et al., 2016), *Osmanthus serrulatus* (Wang et al., 2024), *Phoebe zhennan* (Xiao et al., 2020). In our results, we show that the population C17 located in the northern Sichuan Basin has high genetic diversity and haplotype diversity, and the shared haplotype indicates that the late glacial expansion may have extended from the northern Sichuan Basin where C17 is located to the south. Therefore, we conclude that the first expansion route extends from the north to the south of the Sichuan Basin, which is different from the other two expansion routes.

Therefore, we hypothesized that there were three expansion routes in *P. ternata* during the Holocene, the first from the northern part of the Sichuan Basin to the south, and the other two routes from central and eastern China to the north, respectively. In summary, we believe that *P. ternata* experienced repeated expansion and contraction to participate in the formation of the current distribution pattern.

### 4.4 Management and protection strategy

Maintaining population genetic diversity and intraspecific genetic variation is not only a source of biological evolution but also a prerequisite for the sustainable development of this species and an important part of biodiversity conservation (Liu et al., 2022). In order to protect the population of a species, it is necessary to understand the genetic diversity and population structure of natural populations and protect populations in different regions to increase the genetic links between populations (Shahzad et al., 2017).

*P. ternata* is a unique perennial herb with a variety of medicinal effects and has been used in China for more than 2000 years (Zou et al., 2023). *P. ternata* has high economic value and great market demand. However, in recent years, due to their continuous harvesting and habitat destruction, the natural populations of these *P. ternata* have been greatly depleted. Through the field investigation, it is found that the wild *P. ternata* population is relatively rare, and these important resources need to be protected and managed, so we recommend protecting *P. ternata* in the following four ways. First, on the basis of population genetic analysis, it is suggested that populations with high genetic diversity should be taken as the priority conservation units of this species, such as populations C17, C22, C7, C13, and C19 (Sichuan, Guizhou, Zhejiang, and Jiangxi Provinces), which are located in the Quaternary glaciation refuge and have high genetic diversity, so we should locate these populations in the northern Sichuan Basin. In situ conservation is one of the more effective means to protect organisms. In situ conservation is also commonly used for other plants to protect genetic diversity, such as *Ficus hirta* (Lu et al., 2022); Second, some populations with severe habitat damage can be protected by moving them to more suitable areas for *P. ternata* growth. Third, some artificial means can be used to assist the reproduction of *P. ternata*, such as tissue culture technology and artificial seedling cultivation, so that mature *P. ternata* seeds with high genetic diversity can be artificially planted with other populations to promote gene exchange among populations (Cabrera‐Toledo et al., 2012). Fourth, a combination of information and education to raise public awareness of conservation of species diversity and reduction of habitat fragmentation can control activities that consume population size (e.g., illegal mining) and genetic fragmentation (e.g., habitat loss; Hamilton et al., 2012).

## 5 CONCLUSION

In this study, nine pairs of SSR molecular markers, two cpDNA fragments (*psb*K‐*psb*I and *trn*L‐F) and two pairs of nuclear gene sequences (ITS and ETS) were used to study the genetic diversity, genetic structure, potential dispersal paths, and population dynamic history of more than 270 individuals from 22 *P. ternata* populations. The results of this study indicate that the current systematic geographic pattern and genetic structure of *P. ternata* are affected by climate fluctuations and environmental heterogeneity. The distribution of *P. ternata* in China can be divided into two groups, namely the southwest group and the central and eastern group. Our study also reveals three potential migration routes for *P. ternata* to form the current distribution pattern. The current distribution pattern of *P. ternata* is probably caused by the repeated expansion and contraction of three migration routes during the glacial and interglacial periods. This study can provide scientific theoretical basis for the conservation, development, and utilization of *P. ternata* resources, and even provide a reference for the systematic geographical pattern of large‐scale spatial distribution of plants in China, and enrich our understanding of the evolutionary history of plant species diversity in East Asia.

## AUTHOR CONTRIBUTIONS

**Chunxue Jiang:** Conceptualization (lead); formal analysis (lead); software (lead); writing – original draft (lead); writing – review and editing (lead). **Ming Wu:** Conceptualization (lead); software (equal); supervision (equal). **Shanshan He:** Investigation (equal); software (equal); visualization (equal). **Yuxia Lu:** Methodology (equal); visualization (equal); writing – review and editing (equal). **Cai Zhao:** Investigation (lead); methodology (lead); software (lead); supervision (lead); writing – review and editing (lead).

## FUNDING INFORMATION

Guizhou science and technology support project ([2019]2451‐2), National Natural Science Foundation of China (32260252), Guizhou biology domestic first‐class construction discipline opening fund (GNYL[2017]009), Key Laboratory Opening Project of Education Department of Guizhou Province Ministry of Education (Guizhou Education Cooperation) (KY[2019]033).

## CONFLICT OF INTEREST STATEMENT

The authors declare that there are no competing interests.

## Supporting information


Figure S1.
Figure S2.
Figure S3.
Figure S4.
Figure S5.
Figure S6.

Table S1.
Table S2.
Table S3.
Table S4.
Table S5.
Table S6.
Table S7.
Table S8.
Table S9.

## Data Availability

Some cpDNA, ITS and ETS sequences have been uploaded to NCBI (accession numbers OL310546‐OL310559 and OL310560‐OL310573, PQ163922-PQ163938, PQ186318-PQ186338).
