# Supplementary material for: Phylogeography of a herbal Pinellia ternata reveals repeated range expansions and inter/postglacial recolonization routes on the fragmented distribution pattern in China
Source: Ecol Evol. 2024 Aug 29;14(9):e70206. doi: 10.1002/ece3.70206 (PMC11362505; doi:10.1002/ece3.70206)
Supplement: Supplementary file 1 — Figure S1. Figure S2. Figure S3. Figure S4. Figure S5. Figure S6. [file ECE3-14-e70206-s002.docx]

**Supplementary material**

**Figure S1 Mantel test of genetic distance and geographical distance of *P. ternate***


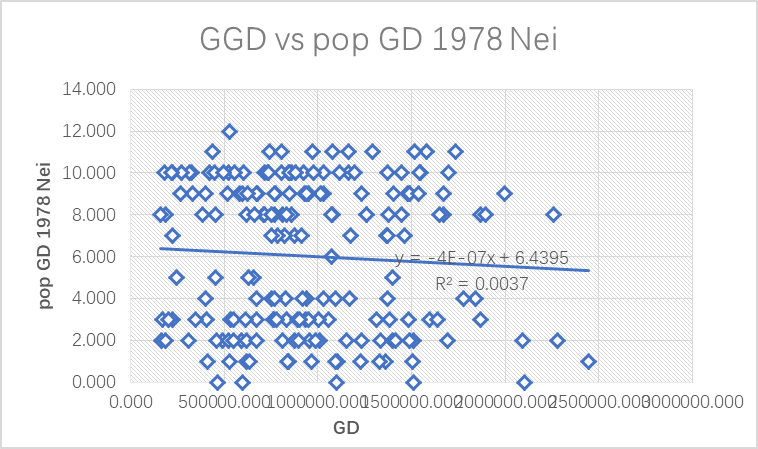


Note: *P*=0.03＜0.05

**Figure S2 NJ tree of *P. ternate* populations based on Nei’s genetic distance**


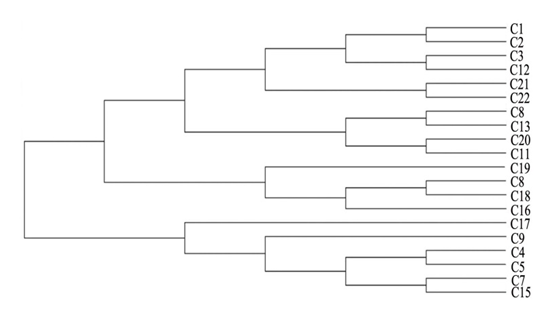


**Figure S3 Results of BARRIER analysis.**

*
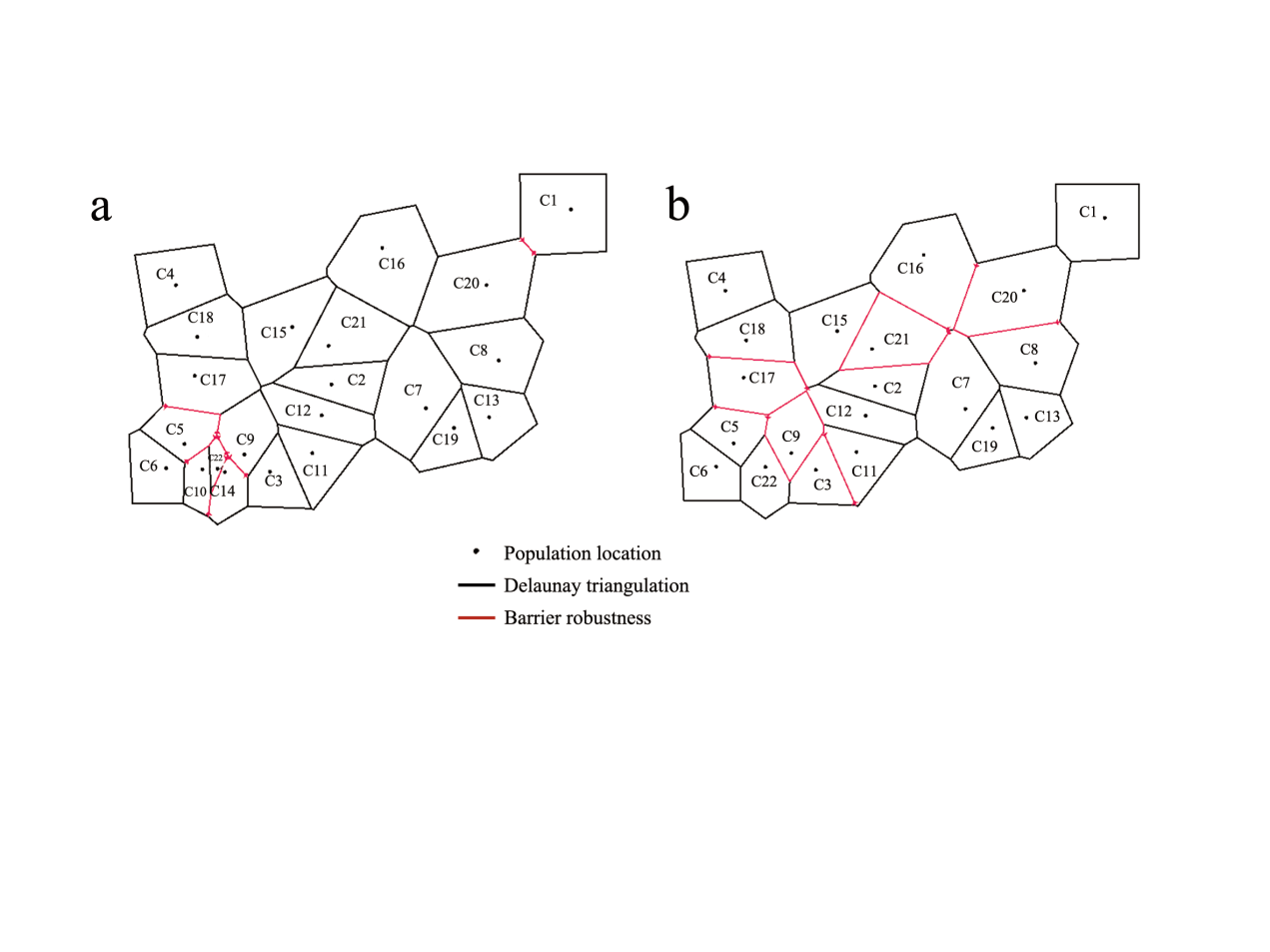
*

a. Barrier analysis results based on cpDNA data; b. Barrier analysis results based on SSR data.

**Figure S4 Mismatch distribution analysis of cpDNA haplotypes for *P. ternate*.**


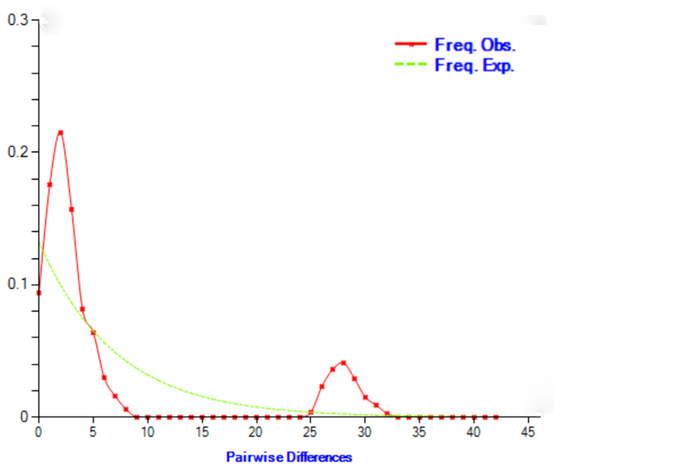


Note: The green line represents the distributions expected for expanding and the red line represents the observed mismatch distribution.

**Figure S5 Mismatch distribution analysis of ETS haplotypes for *P. ternate*.**


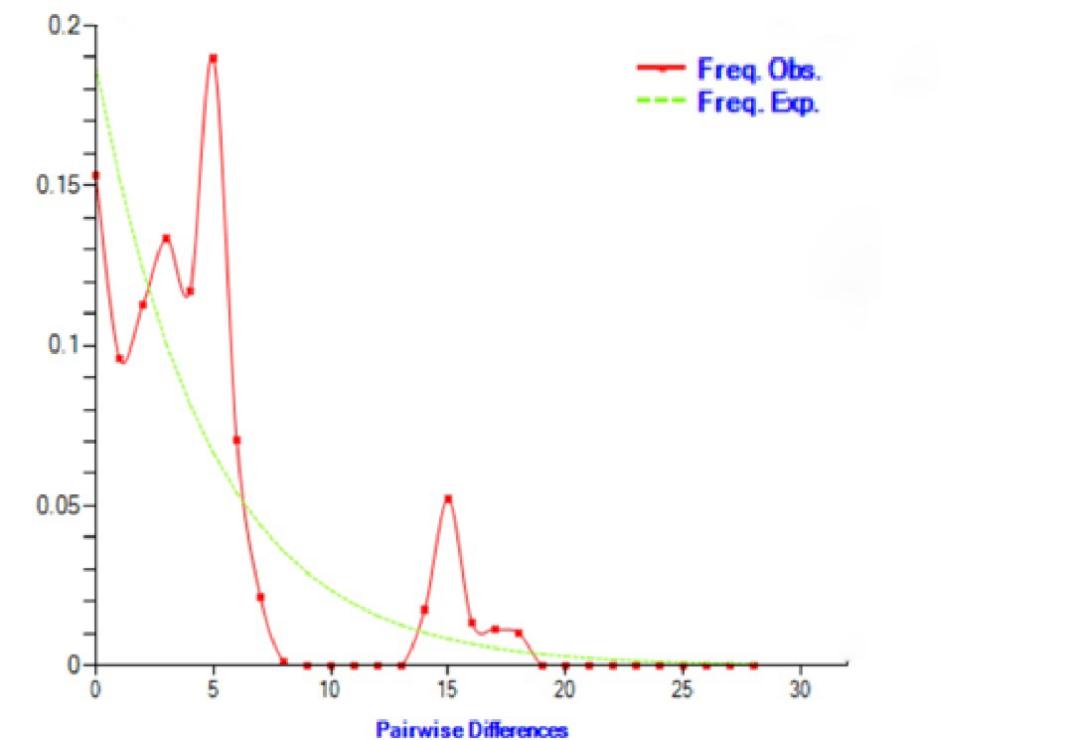


Note: The green line represents the distributions expected for expanding and the red line represents the observed mismatch distribution.

**Figure S6** Mismatch distribution analysis of ITS haplotypes for *P. ternate*.


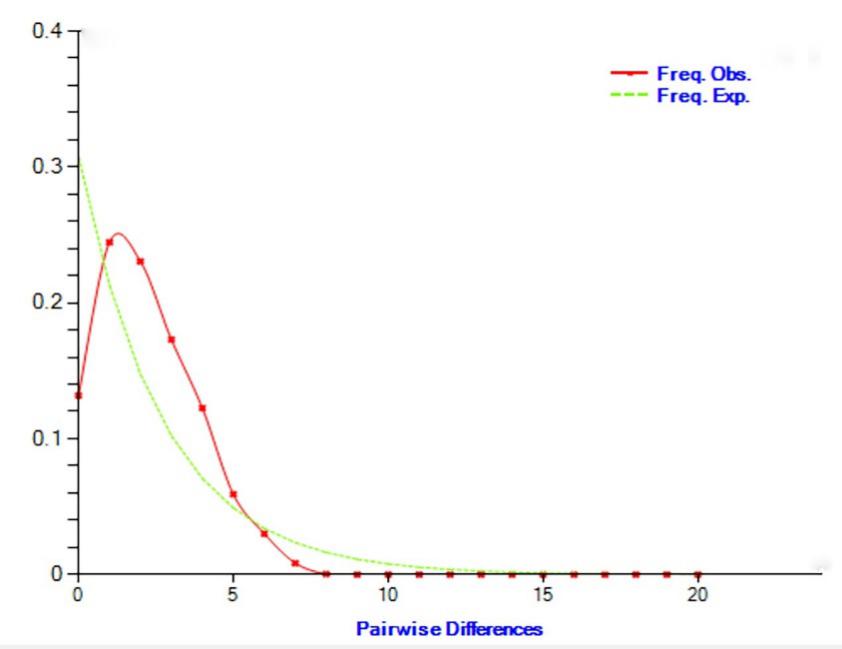


Note: The green line represents the distributions expected for expanding and the red line represents the observed mismatch distribution.
