# Supplementary material for: Phylogeography of a herbal Pinellia ternata reveals repeated range expansions and inter/postglacial recolonization routes on the fragmented distribution pattern in China
Source: Ecol Evol. 2024 Aug 29;14(9):e70206. doi: 10.1002/ece3.70206 (PMC11362505; doi:10.1002/ece3.70206)
Supplement: Supplementary file 2 — Table S1. Table S2. Table S3. Table S4. Table S5. Table S6. Table S7. Table S8. Table S9. [file ECE3-14-e70206-s001.docx]

**Supplementary material**

**Table S1 SSR Geographical distribution of sampled populations of *P. ternata***

| Sample name | Sample size | Location | Longitude (E) | Latitude (N) | Altitude(M) |
| --- | --- | --- | --- | --- | --- |
|  |  |  |  |  |  |
| C1 | 10 | Dandong, Liaoning | 124.3541 | 40.01304 | 22 |
| C2 | 10 | Dangyang, Yichang, Hubei | 111.9413 | 30.92048 | 59 |
| C3 | 10 | Liping, Qiandongnan, Guizhou | 108.7476 | 26.39719 | 644 |
| C4 | 10 | Tianshui, Gansu | 103.8709 | 36.07785 | 1085 |
| C5 | 10 | Zhaotong, Yunnan | 104.3199 | 27.8321 | 897 |
| C6 | 10 | Zhaotong, Yunnan | 103.3744 | 26.5735 | 2142 |
| C7 | 10 | Chizhou , Anhui | 116.8293 | 29.6895 | 34 |
| C8 | 10 | Nantong, Jiangsu | 120.6055 | 32.1733 | 10 |
| C9 | 6 | Kaiyang, Guizhou | 107.4255 | 27.29947 | 1263 |
| C11 | 10 | Shaoyang, Hunan | 110.9461 | 27.36621 | 302 |
| C12 | 10 | Changde , Hunan | 111.4357 | 29.32264 | 68 |
| C13 | 10 | Yiwu , Zhejiang | 120.1234 | 29.22179 | 91 |
| C15 | 6 | Shangluo , Shaanxi | 109.91 | 33.89973 | 702 |
| C16 | 10 | Shijiazhuang ,Hebei | 114.572 | 38.01232 | 75 |
| C17 | 10 | Mianyang , Sichuan | 104.8536 | 31.38133 | 439 |
| C18 | 6 | Longnan, Gansu | 104.9881 | 33.39363 | 898 |
| C19 | 10 | Shangyao, Jiangxi | 118.2978 | 28.67076 | 26 |
| C20 | 10 | Qingdao, Shandon | 119.9825 | 36.08664 | 16 |
| C21 | 10 | Guyuan , Ningxia | 111.7865 | 32.92997 | 1955 |
| C22 | 10 | Bijie, Guizhou | 106.0321 | 26.55288 | 1312 |

**Table S2 Distribution of cpDNA haplotypes and genetic diversity parameters of *P. ternata***

| Population code | cpDNA | | | | |  |
| --- | --- | --- | --- | --- | --- | --- |
|  | n | Pi (×10^-3^) | *Hd* | H | Haplotypes（number） | |
| C1 | 15 | 0 | 0 | 1 | H5(15) | |
| C2 | 15 | 0 | 0 | 1 | H1(15) | |
| C3 | 16 | 0 | 0 | 1 | H1(16) | |
| C4 | 12 | 0 | 0 | 1 | H1(11) | |
| C5 | 10 | 0 | 0 | 1 | H2(10) | |
| C6 | 15 | 0.81 | 0.533 | 2 | H1(7),H2(8) | |
| C7 | 15 | 0.57 | 0.514 | 3 | H1(4),H2(1),H6(10) | |
| C8 | 14 | 0 | 0 | 1 | H1(15) | |
| C9 | 6 | 0 | 0 | 1 | H3(6) | |
| C10 | 6 | 0.81 | 0.803 | 4 | H1 (4),H6(3),H7(2),H10(3) | |
| C11 | 14 | 0 | 0 | 1 | H1(15) | |
| C12 | 12 | 0 | 0 | 1 | H1(12) | |
| C13 | 15 | 0.73 | 0. 0.476 | 2 | H1(5),H1(10) | |
| C14 | 6 | 0.51 | 0.333 | 2 | H1(1),H2(5) | |
| C15 | 6 | 0 | 0 | 1 | H1(6) | |
| C16 | 15 | 0.2 | 0.133 | 2 | H1(14),H2(1) | |
| C17 | 15 | 0.41 | 0.533 | 2 | H1(7),H3(8) | |
| C18 | 6 | 0 | 0 | 1 | H4(6) | |
| C19 | 13 | 0 | 0 | 1 | H1(13) | |
| C20 | 14 | 0 | 0 | 1 | H6(15) | |
| C21 | 14 | 0 | 0 | 1 | H3(14) | |
| C22 | 12 | 1.47 | 0.864 | 7 | H1(3), H2(1), H6(4), H7(1), H8(1), H9(1), H10(1) | |

Note: n: number of sequenced individuals; Pi: nucleotide diversity; Hd: Haplotype diversity index; H: Number of haplotype species.

**Table S3 Distribution and genetic diversity of ITS haplotypes of *P. ternata***

| Population code | ITS | | | | |
| --- | --- | --- | --- | --- | --- |
|  | n | Pi (×10^-3^) | *Hd* | H | Haplotypes（number） |
| C1 | 15 | 0 | 0 | 1 | H1(15) |
| C2 | 15 | 0 | 0 | 1 | H2(15) |
| C3 | 16 | 0.85 | 0.5667 | 3 | H1(2), H3(4), H4(10) |
| C4 | 12 | 0 | 0 | 1 | H3(12) |
| C5 | 10 | 0 | 0 | 1 | H5(10) |
| C6 | 15 | 3.59 | 0.604 | 3 | H5(7), H6(7), H7(1) |
| C7 | 15 | 2.72 | 0.695 | 5 | H6(1),H8(8),H9(1),H10(3),H11(2) |
| C8 | 14 | 0.59 | 0.44 | 2 | H6(4), H12(10) |
| C9 | 6 | 0 | 0 | 1 | H6(6) |
| C10 | 6 | 2.22 | 0.712 | 4 | H6(6), H15(3), H16(1), H17(2) |
| C11 | 14 | 0 | 0 | 1 | H6(14) |
| C12 | 12 | 1.39 | 0.758 | 4 | H6(5), H9(3), H10(3), H13(1) |
| C13 | 15 | 0.33 | 0.248 | 2 | H6(13), H12(2) |
| C14 | 6 | 0 | 0 | 1 | H10(6) |
| C15 | 6 | 0 | 0 | 1 | H14(6) |
| C16 | 15 | 0.64 | 0.476 | 2 | H3(5), H14(10) |
| C17 | 15 | 0.83 | 0.648 | 3 | H1(7), H3(2), H14(6) |
| C18 | 6 | 0 | 0 | 1 | H3(6) |
| C19 | 13 | 0 | 0 | 1 | H6(13) |
| C20 | 14 | 0.71 | 0.527 | 2 | H9(6), H13(8) |
| C21 | 14 | 0 | 0 | 1 | H2(14) |
| C22 | 12 | 2.94 | 0.697 | 4 | H2(5), H6(5), H9(1), H14(1) |

Note: n: number of sequenced individuals; Pi: nucleotide diversity; Hd: Haplotype diversity index; H: Number of haplotype species.

**Table S4 Distribution and genetic diversity of ETS haplotypes of *P. ternata***

| Population code | ETS | | | | |
| --- | --- | --- | --- | --- | --- |
|  | n | Pi (×10^-3^) | *Hd* | H | Haplotypes（number） |
| C1 | 15 | 0 | 0 | 1 | H1(15) |
| C2 | 15 | 0 | 0 | 1 | H2(15) |
| C3 | 15 | 1.81 | 0.51429 | 2 | H3(6),H4(9) |
| C4 | 12 | 0 | 0 | 1 | H5(12) |
| C5 | 10 | 0.94 | 0.53333 | 2 | H5(6),H6(4) |
| C6 | 15 | 0 | 0 | 1 | H5(14) |
| C7 | 15 | 2.41 | 0.71429 | 4 | H5(4),H6(7),H7(3),H8(1) |
| C8 | 14 | 0.93 | 0.26374 | 2 | H5(12),H7(2) |
| C9 | 6 | 1.88 | 0.53333 | 2 | H5(2),H7(4) |
| C10 | 6 | 0 | 0 | 1 | H5(3) |
| C11 | 15 | 2.48 | 0.64762 | 3 | H3(8),H4(4),H5(3) |
| C12 | 12 | 0.85 | 0.48485 | 2 | H5(8),H6(4) |
| C13 | 15 | 0.94 | 0.53333 | 2 | H9(8),H10(7) |
| C14 | 6 | 0 | 0 | 1 | H11(6) |
| C15 | 6 | 0 | 0 | 1 | H12(6) |
| C16 | 15 | 0 | 0 | 1 | H13(15) |
| C17 | 15 | 3.35 | 0.74286 | 4 | H5(5),H14(2),H15(6),H16(2) |
| C18 | 6 | 0 | 0 | 1 | H17(6) |
| C19 | 13 | 0.95 | 0.53846 | 2 | H18(7),H19(6) |
| C20 | 15 | 1.88 | 0.53333 | 2 | H5(8),H7(7) |
| C21 | 14 | 2.32 | 0.61538 | 3 | H16(2),H20(8),H21(4) |
| C22 | 12 | 0 | 0 | 1 | H5(12) |

Note: n: number of sequenced individuals; Pi: nucleotide diversity; Hd: Haplotype diversity index; H: Number of haplotype species.

**Table S5 AMOVA analysis** **based on the cpDNA for populations**

| Source of variation | d. f. | Sum of squares | Variance components | Percentage of variation | Fixation index (*F_ST_*) |
| --- | --- | --- | --- | --- | --- |
| Among populations | 21 | 98.579 | 0.44670 | 77.23* | 0.77233 |
| Within populations | 252 | 35.517 | 0.15049 | 22.77* |  |
| Total | 273 | 134.096 | 0.59719 |  |  |

Note: * indicates a P value less than 0.05.Note: * indicate that P<0.05.

**Table S6 AMOVA analysis based on the ITS for populations**

| Source of variation | d. f. | Sum of squares | Variance components | Percentage of variation | Fixation index (*F_ST_*) |
| --- | --- | --- | --- | --- | --- |
| Among populations | 21 | 223.966 | 0.83923 | 72.12* | 0.72117 |
| Within populations | 250 | 81.119 | 0.32448 | 27.88* |  |
| Total | 271 | 305.085 | 1.16371 |  |  |

Note: * indicate that P<0.05.

**Table S7 AMOVA analysis based on the ETS for populations**

| Source of variation | d. f. | Sum of squares | Variance components | Percentage of variation | Fixation index (*F_ST_*) |
| --- | --- | --- | --- | --- | --- |
| Among populations | 21 | 515.858 | 1.96148 | 86.65* | 0.86646 |
| Within populations | 251 | 75.878 | 0.3023 | 13.35* |  |
| Total | 272 | 591.736 | 2.26378 |  |  |

Note: * indicate that P<0.05.

**Table S8 AMOVA analysis of *P. ternate* population based on SSR**

| Source of variation | d.f. | Sum of squares | Variance components | Percentage of variation | Fixation index （*F*_st_） |
| --- | --- | --- | --- | --- | --- |
|  |  |  |  |  |  |
| Among populations | 19 | 310.113 | 16.322 | 14% | 0.534 |
| Within populations | 188 | 932.5 | 4.96 | 86% |  |

Note: * indicate that P<0.05.

**Table S9 Bottleneck effect analysis of *P. ternate* populations based on three models**

| code | IAM | | | TPM | | | SMM | | |
| --- | --- | --- | --- | --- | --- | --- | --- | --- | --- |
|  | Sign Test | Standardized Differences Test | Wilcoxon Test | Sign Test | Standardized Differences Test | Wilcoxon Test | Sign Test | Standardized Differences Test | Wilcoxon Test |
| C1 | 0.004389 | 0.000086 | 0.001695 | 0.004317 | 0.000456 | 0.001953 | 0.008173 | 0.001953 | 0.001953 |
| C2 | 0.093793 | 0.002808 | 0.019168 | 0.075438 | 0.007438 | 0.006836 | 0.119492 | 0.009766 | 0.018555 |
| C3 | 0.022164 | 0.002769 | 0.060828 | 0.089054 | 0.016468 | 0.004883 | 0.102923 | 0.012207 | 0.052734 |
| C4 | 0.055137 | 0.012308 | 0.105437 | 0.225421 | 0.043956 | 0.027344 | 0.281562 | 0.039063 | 0.054688 |
| C5 | 0.128068 | 0.015808 | 0.170416 | 0.083632 | 0.067082 | 0.125000 | 0.118283 | 0.125000 | 0.150391 |
| C6 | 0.022531 | 0.000306 | 0.005619 | 0.010644 | 0.002237 | 0.002441 | 0.015594 | 0.006836 | 0.009277 |
| C7 | 0.061549 | 0.002801 | 0.031977 | 0.062156 | 0.011805 | 0.006836 | 0.095229 | 0.012207 | 0.016113 |
| C8 | 0.276316 | 0.157149 | 0.285823 | 0.60385 | 0.397869 | 0.248047 | 0.612353 | 0.367188 | 0.673828 |
| C9 | 0.042064 | 0.001659 | 0.020590 | 0.080675 | 0.006996 | 0.004883 | 0.142423 | 0.004883 | 0.016113 |
| C11 | 0.003927 | 0.000698 | 0.025414 | 0.034448 | 0.005115 | 0.000977 | 0.056056 | 0.001953 | 0.002930 |
| C12 | 0.244824 | 0.024400 | 0.204698 | 0.240091 | 0.081883 | 0.019531 | 0.286513 | 0.125000 | 0.273438 |
| C13 | 0.026380 | 0.000517 | 0.005112 | 0.031531 | 0.002159 | 0.003906 | 0.050936 | 0.009766 | 0.013672 |
| C15 | 0.004460 | 0.00014 | 0.001348 | 0.009351 | 0.000534 | 0.003906 | 0.012480 | 0.003906 | 0.003906 |
| C16 | 0.018175 | 0.00355 | 0.089192 | 0.253589 | 0.02346 | 0.000977 | 0.561911 | 0.012207 | 0.137695 |
| C17 | 0.153516 | 0.050177 | 0.364800 | 0.140288 | 0.151144 | 0.150391 | 0.408760 | 0.248047 | 0.285156 |
| C18 | 0.072591 | 0.02004 | 0.116333 | 0.480011 | 0.055839 | 0.013672 | 0.508170 | 0.037109 | 0.125000 |
| C19 | 0.031012 | 0.005979 | 0.137417 | 0.036044 | 0.036135 | 0.001953 | 0.411913 | 0.009766 | 0.082031 |
| C20 | 0.008899 | 0.001442 | 0.034547 | 0.064912 | 0.008369 | 0.001953 | 0.266284 | 0.005859 | 0.013672 |
| C21 | 0.001641 | 0.000120 | 0.003294 | 0.019450 | 0.000652 | 0.000977 | 0.039481 | 0.001953 | 0.001953 |
| C22 | 0.038468 | 0.003787 | 0.082847 | 0.151132 | 0.021957 | 0.001953 | 0.390773 | 0.006836 | 0.082031 |
